# Control strategies for underactuated neural ensembles driven by optogenetic stimulation

**DOI:** 10.3389/fncir.2013.00054

**Published:** 2013-04-09

**Authors:** ShiNung Ching, Jason T. Ritt

**Affiliations:** ^1^Department of Electrical and Systems Engineering, Washington University in St. LouisSt. Louis, MO, USA; ^2^Department of Biomedical Engineering, Boston UniversityBoston, MA, USA

**Keywords:** control, stimulation, neuroprosthetics, optogenetics, cortex, computational neuroscience, dynamical systems

## Abstract

Motivated by experiments employing optogenetic stimulation of cortical regions, we consider spike control strategies for ensembles of uncoupled integrate and fire neurons with a common conductance input. We construct strategies for control of spike patterns, that is, multineuron trains of action potentials, up to some maximal spike rate determined by the neural biophysics. We emphasize a constructive role for parameter heterogeneity, and find a simple rule for controllability in pairs of neurons. In particular, we determine parameters for which common drive is not limited to inducing synchronous spiking. For large ensembles, we determine how the number of controllable neurons varies with the number of observed (recorded) neurons, and what collateral spiking occurs in the full ensemble during control of the subensemble. While complete control of spiking in every neuron is not possible with a single input, we find that a degree of subensemble control is made possible by exploiting dynamical heterogeneity. As most available technologies for neural stimulation are underactuated, in the sense that the number of target neurons far exceeds the number of independent channels of stimulation, these results suggest partial control strategies that may be important in the development of sensory neuroprosthetics and other neurocontrol applications.

## 1. Introduction

Neurocontrol underlies an expanding range of applications, especially in the development of neuroprosthetics (Shenoy et al., 2003). By “neurocontrol” we here mean control of neural systems, as distinct from control of external devices via decoding of recordings of neural activity, as is most common in brain machine interfaces (Lebedev and Nicolelis, 2006). Neurocontrol, in principle, is the application of established control theory to the stimulation of neural tissue (Khalil, 2002; Danzl et al., 2009; Schiff, 2009; Liu et al., 2010; Ahmadian et al., 2011; Dasanayake and Li, 2011). However, the scale and complexity of nervous systems, coupled with the stochastic nature of biological function, suggests that a different attitude and problem definition from traditional control theory may be appropriate. Most notably, the dynamics in neuronal networks are highly non-linear and complex, involving a large number of neurons, so that generically we expect radical underactuation: there are orders of magnitude fewer independent control inputs than degrees of freedom in the system.

Compared to electrical microstimulation, optogenetic stimulation (Zhang et al., 2009; Anikeeva et al., 2011; Deisseroth, 2011; Peron and Svoboda, 2011; Siegle et al., 2011; Yizhar et al., 2011) does not overcome the underactuation issue, but does provide some new opportunities in control design. One is cell-type specificity, which allows for distinct control of different components of a neural circuit, raising the question of which target best controls the overall network behavior. Optogenetics also employs a different mechanism of perturbation, namely modulation of ion channel conductance rather than direct current injection, and can provide hyperpolarizing as well as depolarizing stimulation. A somewhat less appreciated aspect of optogenetic stimulation is that broader areas may be activated per input than with electrical microstimulation, due to the ability to illuminate larger tissue volumes. In the context of a single or small number of illumination sources, common in experiments using chronic implants in behaving animals, stimulation might be thought to produce only bulk, synchronous firing: a mass of action potentials induced with each stimulating pulse.

In classical control theory, the notion of controllability relates to the ability to determine the exact trajectory of a dynamical system (Khalil, 2002). Given the challenges outlined above, for a neuronal system this objective is clearly difficult. The goal of this paper is to consider weaker controllability goals, which we believe are more applicable to underactuated neurocontrol situations. We use the simple integrate and fire model to determine conditions for spike sequence controllability in pairs of neurons, driven by a common light source, and use the pairwise results to understand the limits of control in large uncoupled ensembles under bulk illumination. In particular, we argue that although independent control of individual neurons is limited, exploitation of cellular heterogeneity in membrane charging rates and optogenetic expression nevertheless allows a significant degree of independence in imposed spike patterning.

## 2. Results

### 2.1. Control formulation appropriate for ensemble stimulation

**Figure 1:**
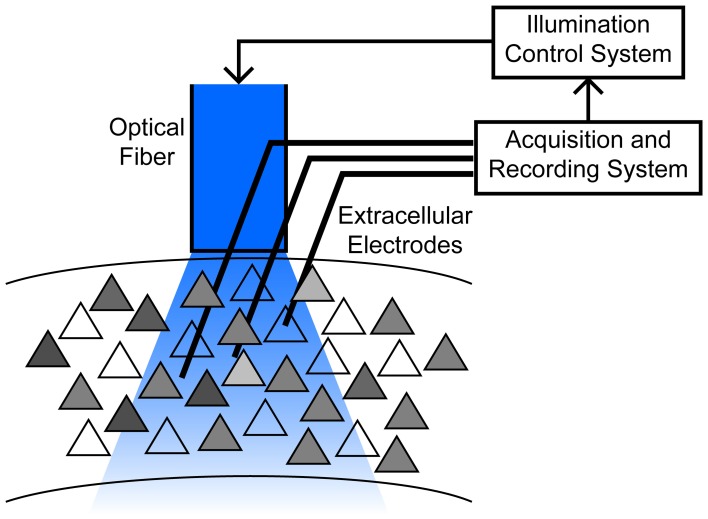
**Schematic of the motivating application, typical of chronic implants in freely moving animals.** An optical fiber is chronically implanted above a cortical area containing hundreds to thousands of neurons with varying degree of ChR2 expression (*gray shading*). Responses to stimulation are heterogeneous due to these expression differences, as well as their location relative to the fiber, cell properties such as electrotonic size and other channel densities, etc. Multiple electrodes penetrate into a broad region illuminated by the fiber, permitting observation of a small subset of neurons. See (Siegle et al., 2011) for example methodology.

Massive underactuation is typical of neurocontrol problems. At the heart of the challenge is the inability to “address” individual neurons with separate time varying control inputs. Within a domain surrounding a single stimulating electrode, there may be hundreds or more of neurons, of different cell types, and in typical optogenetic settings, e.g., with a large (200 μm) fiber sitting hundreds of micrometers away from the target population, illumination can spread over even larger regions (Figure 1). Possible improvements, such as laser scanning systems, are limited by requirements to immobilize the head, and preferentially actuate shallow tissue depths due to light absorption and scattering (Peron and Svoboda, 2011; Yizhar et al., 2011). Compounding the difficulty are constraints on the input form—light intensity can be modulated to induce different levels of ChR2 activation, but a negative input requires a distinct genetic manipulation, such as insertion of Halorhodopsin or ArchT (Fenno et al., 2011). An additional complication in experimental applications is that the spiking activity of only a fraction of neurons in a given area is directly observed, so control strategies for a large ensemble treats recorded neurons as proxies for the general network.

We thus posit that few, if any, neuronal networks will be controllable under the classical engineering definition that *all* states (leading to spike sequences) are reachable from *any* starting condition under a physically realizable control (Khalil, 2002). With such a definition, it might be thought that controllability is simply not attainable. To some extent this is true, as we show with a severely limiting necessary condition below. We instead look for less restrictive notions of controllability that are still sufficiently useful in neural applications. We use the following definitions:

**Definition 1** (Spike pattern). *Consider an ensemble of *N* neurons, labeled* {1, 2, …, *N*}. *A sequence of pairs of labels and times*, 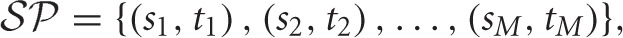 *where*

$$s_{k}\in \{1,\;2,\;\ldots ,\;N\}\text{, }and\;t_{1}\leq t_{2}\leq \ldots \leq t_{M}\;are\;real\;numbers,$$

*is a spike pattern. A pair (*s*_*k*_, *t*_*k*_) represents a spike from neuron *s*_*k*_ at time *t*_*k*_*.

**Definition 2** (Spike sequence). *For the same ensemble, a sequence of labels without timing information*,

$$S=\left\{s_{1},\;s_{2},\;\ldots ,\;s_{M}\right\},$$

*is a spike sequence*.

**Definition 3** (Controllability). *An ensemble is sequence/pattern controllable if all possible sequences/patterns can be achieved. That is, for any sequence/pattern S and any initial conditions, there exists an input that results in S*.

An important special case of sequence controllability is the ability to control which neurons spike following a baseline condition, for example at trial onsets in a behavioral experiment. We are here defining only deterministic spiking control, but consider noise in simulation below.

The definitions suggest at least three ways in which the notion of controllability could be relaxed in underactuated applications: (i) we accept that some spike sequences or patterns are not achievable, and concentrate only on an achievable subclass (with the hope that this class contains a large enough set of sequences of functional relevance); (ii) we accept that in a typical ensemble, some fraction of the neurons will not be controllable, and concentrate on a subensemble of neurons that is (and check if activity in the uncontrolled neurons disrupts the function we are attempting to control); or (iii) we accept that there will be limits to the precision of the temporal structure we can attain, but try to achieve patterns “close” to desired patterns.

Previous studies (McIntyre and Grill, 1999; Liu et al., 2010, 2011; Ahmadian et al., 2011) mainly focus on option (iii) and try to limit how bad “close” can be. We here concentrate on the first two options, starting with sequence controllability, without concern for timing. It is inherent to underactuated systems that spike patterns that are too fast will not be achievable. However, if the desired spike timing is slow enough, sequence controllability is sufficient for pattern controllability, and the issue becomes a quantitative and application specific question of what counts as fast enough (see Discussion). In many cases, it may be more important to selectively activate subsets of neurons rather than sequences *per se*. Again thinking of a behavioral task, if the ensemble is sequence controllable, then it is also possible to activate different neurons according to trial type, for example to assess the animal's perceptual sensitivity to different subpopulations [contrasted with assessing sensitivity only to the total number of activated neurons (Huber et al., 2008)]. We stress that, in contrast to approaches minimizing an objective function that combines spike timing precision with errors for added or missed spikes (Ahmadian et al., 2011), our approach is to accept the existence of a maximum spike rate, and achieve controllability below this rate, consistent with approach (i). Once this rate is determined, it is sufficient to establish sequence controllability. We address (ii) by determining what conditions are required for a neuron pair to be controllable, and how the pairwise result constrains controllable subensembles within a large population.

### 2.2. IAF neural model

We will consider ensembles for which we have a single one dimensional control input, although the effect of this input on each cell may differ in magnitude or algebraic form. Our choice is motivated by experiments (Figure 1) in which we deliver illumination to a cortical region through a single, fixed optical fiber, driving a population of hundreds to thousands of neurons expressing Channelrhodopsin-2 (ChR2) (Deisseroth, 2011; Siegle et al., 2011). To focus on ensemble properties, we analyze a highly simplified neuron model, the Integrate and Fire neuron (*IAF*) (Dayan and Abbott, 2005). Consider a noiseless, uncoupled ensemble where the *kth* neuron is described by the IAF differential equation:

(1) $$\frac{dv_{k}}{dt}=-\alpha_{k}v_{k}+g_{C}(t)\beta_{k}\left(E-v_{k}\right)$$

where *v*_*k*_ is the membrane potential, and α_*k*_ is the decay rate of the neuron (the reciprocal of the passive membrane time constant). Coupled to Equation (1) is a reset mechanism whereby a spike is elicited when the membrane potential reaches some threshold, *v*_*k*_ = *v*_*T*_, and the state is reset to *v*_*k*_ = 0. The optogenetic control input is the conductance *g*_*C*_(*t*), which must be non-negative, in the second term. For ChR2 we assume *E* > *v*_*T*_, and *E* takes the same value across all neurons.

The critical assumption is that *g*_*C*_(*t*) is the same for all cells in the network, capturing the experimental configuration of a single optical fiber illuminating a relatively large number of cells (Figure 1). Variation in responsiveness to the light is captured by the parameters β_*k*_, which will depend on the neuron's location relative to the light source, expression levels of ChR2, and other factors. We will find by analysis of the dynamics that the limitation of common temporal input is to some degree overcome by the heterogeneity of parameters (β_*k*_, α_*k*_). The simplicity of the model will make derivation of controllability conditions and extension to larger ensembles straightforward. Equation (1) defines a bilinear control problem, for which some general results are available (see Discussion). We will follow a more direct, constructive approach without attempting to find an optimal control solution.

### 2.3. Sequence controllability in two neurons

We first consider pairwise control of spike sequences. We assume a heterogeneous pair, that is, α_1_ ≠ α_2_ and β_1_ ≠ β_2_. We choose to label the neurons such that α_1_ > α_2_, meaning neuron 1 is more “leaky” than neuron 2. We start with the clear proposition that if one cell is both more weakly driven and more leaky, then (outside a small set of initial conditions) it cannot be made to spike without first inducing a spike in the other cell.

**Proposition 1**. *(Necessary condition) The ensemble Equation* (1) *with *N* = 2 is sequence controllable only if*

(2) $$\frac{\alpha_{1}-\alpha_{2}}{\beta_{1}-\beta_{2}}>0.$$

Given our α_*k*_ label assumption, this condition is equivalent to β_1_ > β_2_. In other words, the differences in decay rates and optogenetic drive must have the same sign.

Suppose otherwise. Then if at any time *v*_1_ = v_*_ = *v*_2_, for some 0 ≤ v_*_ < *E*, we have for all *g*_*C*_ ≥ 0,

$$\frac{dv_{1}}{dt}<\frac{dv_{2}}{dt}.$$

Hence, there is no non-negative control *g*_*C*_(*t*) that allows *v*_1_ to “cross” *v*_2_ from a lower to higher voltage. This crossing is required in any sequence of the form

$$S=\left\{s_{1},\;s_{2},\;\ldots ,\;s_{k},\;1,\;1,\;s_{k+1},\ldots \right\},$$

that is, in any sequence in which there are two or more spikes from cell 1 without a spike from cell 2 in-between.

Proposition 1 provides a necessary condition for sequence controllability, and suggests a pessimistic outcome for even mild underactuation. For a large set of parameters, arbitrary sequences are not achievable. If pairs are selected randomly from a heterogeneous ensemble, the probability that the pair is controllable could be anywhere from zero to one depending on the joint distribution of α and β. For generic distributions, the probability should be near 1/2, given the symmetry of Equation (2). Much of our later ensemble analysis is motivated by what is achievable in spite of this negative condition. To further explore these limits, we establish specific control strategies. In the positive direction, we have

**Proposition 2**. *(Sufficient condition) The ensemble Equation* (1) *with *N* = 2 is sequence controllable if, in addition to Equation* (2),

(3) $$\frac{\alpha_{1}}{\beta_{1}}>\frac{\alpha_{2}}{\beta_{2}}$$

The proof of the sufficient condition is more involved than for the necessary condition, and we follow a constructive approach, without specifically seeking an optimal control. We first simplify the analysis by re-scaling the equations with the substitutions *t* = α_1_ τ, $\hat{\beta }=\beta_{2}/\beta_{1}$, $\hat{\alpha }=\alpha_{2}/\alpha_{1}$, and *g*(*t*) = (β_1_/α_1_)g_*C*_(*t*) to get

(4) $$\frac{dv_{1}}{d\tau }=-v_{1}+g(\tau )(E-v_{1})$$

(5) $$\frac{dv_{2}}{d\tau }=-\hat{\alpha }v_{2}+\hat{\beta }g(\tau )(E-v_{2}).$$

**Figure 2:**
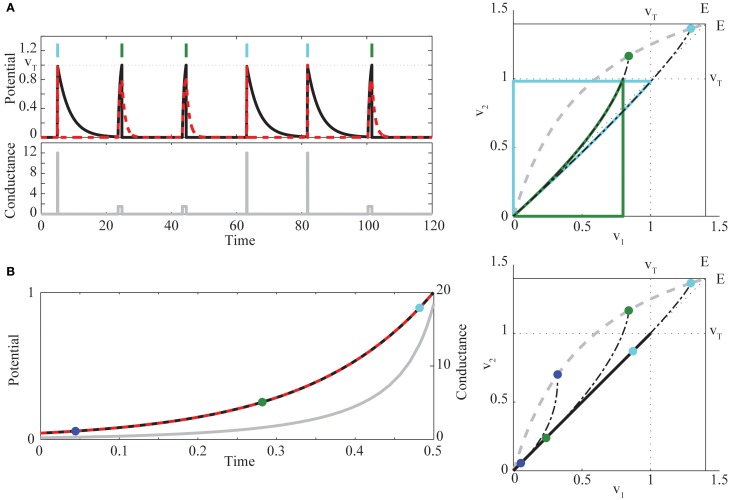
**(A)** (*left*) Example of bimodal pulse control in two neurons, for the target sequence (1, 2, 2, 1, 1, 2), indicated by *light blue* (neuron 1) and *green* (neuron 2) vertical bars [*red dashed*, *v*_1_(*t*), *black*, *v*_2_(*t*), *gray*, *g*(*t*)]. (*right*) Quasi-static phase plane illustrating bimodal control trajectories. For each fixed value of *g*, there exists a unique stable fixed point *V*_*eq*_ lying on a curve (*dashed gray*) running from (0, 0) to (*E*, *E*). The two chosen values of conductance *g*_*k*_ determine if the solution starting at the origin first hits the threshold for neuron 1 (*light blue* trajectory, *g*_1_ = 12) or neuron 2 (*green* trajectory, *g*_2_ = 1.5). Trajectories are continued through reset and return to rest, when *g*(*t*) = 0 after the spike. Also shown (*dots*) are the fixed points *V*_*eq*_(*g*_*k*_), and the trajectories that would be followed without reset if *g*(*t*) were held at the values *g*_*k*_ (*black dot-dashed* curves). **(B)** (*left*) Example of synchronous control. Colors are as in **(A)**. For comparison to the phase plane (*right*), the times at which *g* = (0.3, 1.5, 12) are marked with *blue*, *green*, and *light blue* dots. At these times, the trajectory from the common voltage towards *V*_*eq*_(*g*) is tangent to the diagonal. Parameters chosen for visualization are $\hat{\alpha }=0.27$, β = 0.9, and *E* = 1.4.

We proceed with a quasi-static phase plane analysis, as illustrated in Figure 2.

For each constant *g* there exists a unique, stable fixed point of the ODEs Equations (4)–(5) without reset, at

(6) $$V_{eq}=\left(\begin{array}{c} \frac{gE}{1+g}\\ \frac{gE}{\hat{\alpha }/\beta +g} \end{array}\right).$$

The curve of fixed points *V*_*eq*_(*g*) runs from (0, 0) to (*E*, *E*) as *g* increases, above or below the diagonal according to whether $\hat{\alpha }=\alpha_{2}/\alpha_{1}$ is less or greater than $\hat{\beta }$. When *V*_*eq*_ lies outside the square with corners (0, 0) and (*v*_*T*_, *v*_*T*_), at least one of the neurons will cross the spiking threshold *v*_*T*_. The conditions α_1_ > α_2_ and Equation (2) yield $0<\hat{\alpha },\;\hat{\beta }<1$, while the additional condition Equation (3) is equivalent to $\hat{\alpha }<\hat{\beta }$. All trajectories converge on *V*_*eq*_ proportional to exp{−(1 + *g*)τ} in *v*_1_ and $\exp\{-(\hat{\alpha }+\hat{\beta }g)\tau \}$ in *v*_2_. Hence, the fixed point has “fast” horizontal and “slow” vertical eigenvectors ($\hat{\alpha }+\hat{\beta }g<1+g$ for all *g*), and trajectories converge asymptotically to the fixed point, with trajectory tangent to the vertical eigenvector. The eigenvector geometry manifests in concave upward voltage trajectories in the phase plane. It is this curvature, combined with the fixed point locations relative to the spike thresholds, that underlies the condition Equation (3).

To see this, we construct an explicit control policy assuming Equation (3), so that *V*_*eq*_(*g*) lies above the diagonal (see Figure 2A *right*). For an intermediate range of *g*, the equilibrium voltages satisfy $gE/(1+g)<v_{T}<gE/(\hat{\alpha }/\hat{\beta }+g)$, and spiking is possible only for neuron 2. At high enough *g*, both neurons can spike. However, for many initial conditions, and in particular with both neurons starting near rest, the trajectory for high *g* will cross *v*_*T*_ in the *v*_1_ direction before the *v*_2_ direction, due to the upward curvature. If $\hat{\alpha }=\alpha_{2}/\alpha_{1}$ and $\hat{\beta }$ are known, we can therefore construct a simple control strategy for sequences, employing pulsed *g* with two different amplitudes: *g*_2_ is chosen so that *V*_*eq*_(*g*_2_) lies in the region above *v*_2_ = *v*_*T*_ but left of *v*_1_ = *v*_*T*_. Then *g*_1_ > g_2_ is chosen so that *V*_*eq*_(*g*_1_) is between (*v*_*T*_, *v*_*T*_) and (*E*, *E*) and that, in backwards time, the trajectory through (*v*_*T*_, *v*_*T*_) hits the *v*_2_ axis above the origin. Such a trajectory exists when the slope through (*v*_*T*_, *v*_*T*_) is less than one, which direct calculation shows is the case for high enough *g*_1_ (when Equation (2) is satisfied). For this choice, any trajectory starting near rest will hit *v*_1_ = *v*_*T*_ before hitting *v*_2_ = *v*_*T*_. Thus for the pair of neurons, applying *g*(*t*) = *g*_*k*_, *k* = 1, 2, will generate a spike from neuron *k* before a spike in the other neuron. After the spike, we can apply *g*(*t*) = 0 long enough that both neurons return near rest (e.g., for five times the longest decay time constant), and then apply the appropriate *g*_*k*_ for the next spike in the sequence. This waiting time is what imposes a maximal rate on our control; in the Discussion we place this rate in the context of observed time constants in real neurons. Figure 2A provides an example of this strategy, and illustrates the corresponding phase plane geometry. Intuitively, we find that the cell with larger leak (α_1_) but higher light sensitivity (β_1_) is activated first by large, transient light pulses, whereas the cell with lower light sensitivity (β_2_) but smaller leak (α_2_) can be activated by longer, smaller amplitude light pulses that leave neuron 1 subthreshold. The condition Equation (3) ensures that the quantitative tradeoff between the amount of membrane charging required to reach threshold and the size of the optogenetic current allows the more leaky cell to “win the race to threshold” for large pulses (when both neurons can spike), while in general requiring more light to reach threshold than neuron 2. This proves the sufficiency of Equation (3).

To extend the pairwise result to large ensembles, it will be useful to employ an alternative idealized strategy that brings the two neurons synchronously to spike threshold. Consider a pair of neurons at a common voltage *v*_1_ = v_*_ = *v*_2_. Define g_*_ as the conductance for which the difference η(*t*) ≡ *v*_2_(*t*) − *v*_1_(*t*), at this common voltage, has zero temporal derivative, yielding

(7) $$g_{*}\equiv \left(\frac{\alpha_{2}-\alpha_{1}}{\beta_{2}-\beta_{1}}\right)\frac{v_{*}}{E-v_{*}}>0.$$

where, for 0 < v_*_ < *E*, the inequality follows from Equation (2). To elicit a spike we need that common voltage to be increasing. Inserting g_*_ into Equation (1) shows again the sufficiency of Equation (3), in making both right hand sides (for *k* = 1, 2) positive. Figure 2B illustrates this control algorithm. As a technical point, under this policy the origin is an unstable equilibrium. A short, low amplitude pre-pulse in *g*, or the presence of small noise in the system, is thus necessary to bring the neurons away from the origin before carrying them along the diagonal according to Equation (7).

### 2.4. Geometric interpretation of conditions

**Figure 3:**
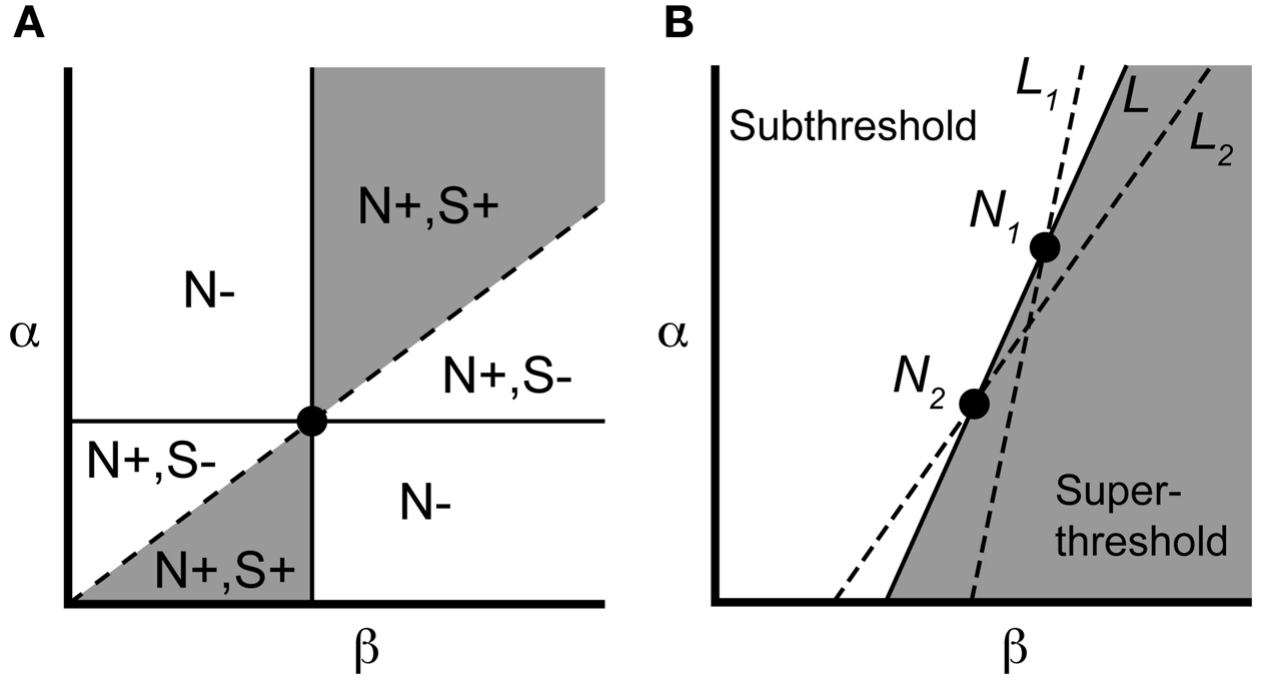
**Parameter space visualization of control approach. (A)** A given neuron, identified by its values for α and β (*dot*), will be pairwise controllable with any neuron satisfying the necessary [*N+*; Equation (2)] and sufficient [*S+*; Equation (3)] conditions (region shown in *gray*). **(B)** Two neurons *N*_1_ and *N*_2_ can be made to spike simultaneously under the input Equation (7), along with any neuron lying along the line *L* (see text). Neurons to the left of *L* will remain subthreshold, while neurons to the right will spike. If we instead choose input Equation (7) corresponding to lines *L*_1_ or *L*_2_, we selectively elicit a spike from neuron 1 or 2, respectively, along with overlapping but distinct subensembles of neurons (to the right of the chosen line).

We can concisely summarize the above results through geometric constructions on the parameters. If we identify neurons by their location in the (β, α) parameter plane (Figure 3), the necessary condition Equation (2) requires the line connecting a controllable pair to have positive slope. Recalling the pairwise labeling convention α_1_ > α_2_, the sufficient condition Equation (3) says the line from the origin to (β_1_, α_1_) should lie above the line from the origin to (β_2_, α_2_). The gray region in Figure 3 indicates that a particular neuron can be in a controllable pair with a limited but still significant range of other neurons.

Now consider a controllable pair, denoted *N*_1_, *N*_2_ in Figure 3B. For *N*_1_, *N*_2_ having equal initial conditions, the input g_*_ causes the two neurons to spike simultaneously at a time denoted *T*_*s*_. It follows from the form of Equation (7) that any neuron that is collinear with *N*_1_ and *N*_2_ in the (β, α)-plane also reaches threshold at time *T*_*s*_ (starting from rest) since the leading term

(8) $$\frac{\left(\alpha_{1}-\alpha_{2}\right)}{\left(\beta_{1}-\beta_{2}\right)}$$

defines the slope of the line *L* connecting *N*_1_, *N*_2_ (Figure 3B). By monotonicity of the IAF model, any neuron that lies to the right of *L* will have hit threshold before time *t* = *T*_*s*_, since it possesses a larger channel conductance β_*k*_ relative to α_*k*_. Similarly, any neuron that lies to the left of *L* is subthreshold at *t* = *T*_*s*_. More generally, every line *L* of positive slope in the (β, α) plane defines a control, via Equation (7), whose application splits the ensemble into silent neurons (to the left) and spiking neurons (to the right and including *L*). For a pair of neurons, constructing the selective control is equivalent to picking one of two lines. In particular, we can choose the line *L*_2_ in Figure 3 to make cell *N*_2_ spike (simultaneously with any other cells along *L*_2_), while cell *N*_1_ will remain subthreshold. Conversely, the line *L*_1_ elicits a spike in *N*_1_ while keeping cell *N*_2_ subthreshold. We will use this geometric interpretation of the applied control to select controllable subensembles, and assess collateral spiking, in large ensembles.

### 2.5. Conductance based neural models

**Figure 4:**
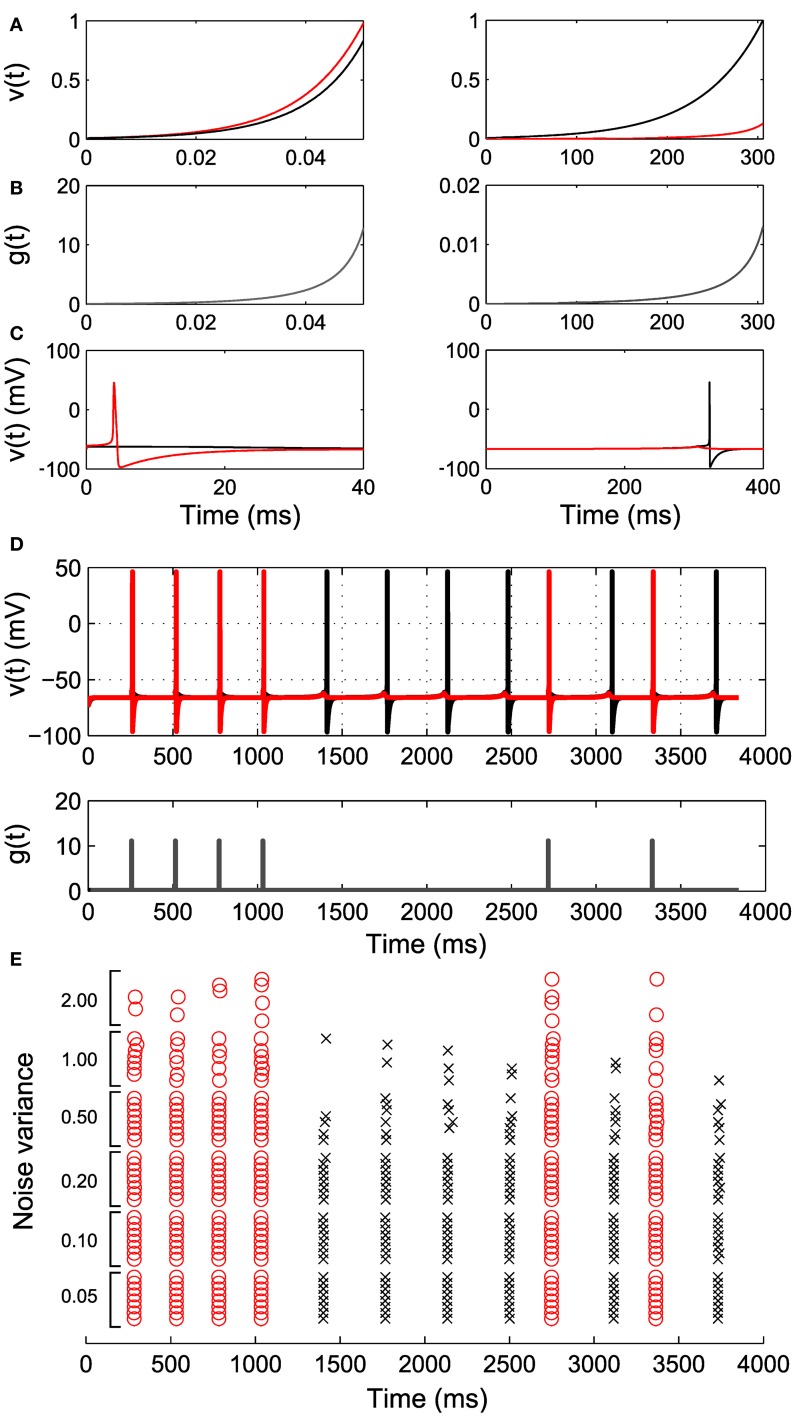
**Simulation of Hodgkin–Huxley-type neurons.** Two integrate and fire neurons are fit to the HH neurons and control inputs are derived according to our algorithm. **(A)** The response of the integrate and fire neurons to the derived control inputs in **(B)**. The red trace corresponds to cell with larger channel conductance. **(C)** The response of the original HH neurons to the derived control inputs, illustrating differential spiking. **(D)** An example spike sequence (111122221212) achieved by interleaving the derived inputs. Note the difference in magnitude and duration of *g*(*t*) in **(B)**. **(E)** Effect of noise of increasing variance on the achieved sequence. Eight realizations at each noise level are shown.

Before turning to large ensembles, we illustrate the two cell case for more realistic neural dynamics with a Hodgkin–Huxley type model with explicit conductance-based spike mechanism (Ching et al., 2010, 2012). We fit “proxy” IAF neurons to the subthreshold voltage of the two neurons, and use the parameters of these proxy neurons to construct the control *g*(*t*) (Figures 4A–C; see Methods). Figure 4D shows successful sequence controllability in this more realistic neural model, despite the control construction depending on the approximating IAF dynamics.

Another important factor in real neurons is noise. In Figure 4E we show that sequence controllability persists in the presence of noise. As the noise level is increased, we begin to lose spikes at desired times; the control *g*(*t*) is constructed to increase for exactly the time required to spike under the deterministic dynamics, and does not overshoot to account for noise-induced lags. There is also a small increase in spike time jitter. However, in general this simplest approach to control of more realistic neural models continues to work well up to moderate noise levels. Improving the robustness of this approach in more general neural models is a subject of ongoing study.

### 2.6. Control of subensembles

Having established pairwise sequence controllability using the input (7), we now expand the approach to large ensembles. Returning to the motivating problem schematized in Figure 1, we imagine we are illuminating an ensemble of ChR2 positive neurons, with multiple electrodes within the region (e.g., using multiple tetrodes Siegle et al., 2011, or an implanted electrode array Song et al., 2009). We suppose we have some number of “recorded” neurons, for which (β, α) are known, as if determined by a previous system identification step using nearby electrodes. We suppose that only a small fraction of cells are recorded within the illuminated area.

As before, we identify the *k*th neuron by its parameters (β_*k*_, α_*k*_). Within the full ensemble, we want to construct a subensemble $N_{M}$ that is sequence controllable, that is, a subset of *M* neurons that can be activated in an arbitrary sequence by application of a dynamic stimulus. We choose the labeling such that

(9) $$\beta_{0}<\beta_{1}<\;\cdots \;<\beta_{N}.$$

Then in order for $N_{M}$ to be pairwise controllable, for all (α_*k*_, β_*k*_), $(\alpha_{l},\;\beta_{l})\in N_{M}$, *k* < l, we need to select neurons such that

(10) $$\begin{array}{l} (\text{i})\alpha_{k}<\alpha_{l}\\ (\text{ii})\alpha_{k}/b_{k}<\alpha_{l}/b_{l}. \end{array}$$

However, pairwise controllability does not guarantee controllability of $N_{M}$. Recalling Figure 3, we must be able to choose, for each neuron in $N_{M}$, a corresponding line that splits the (β, α) plane with our target cell to the right, and the rest of $N_{M}$ to the left. This amounts to the neurons in $N_{M}$ satisfying an additional convexity constraint,

(11) $$\left(\alpha_{1}-\alpha_{0}\right)/\left(\beta_{1}-\beta_{0}\right)<\left(\alpha_{2}-\alpha_{1}\right)/\left(\beta_{2}-\beta_{1}\right)<\;\ldots ,$$

which states that the associated control lines must increase in slope from the left-most to right-most neuron in $N_{M}$. In other words, the collection of lines bound a convex region in the (β, α) plane. For any reasonable distribution of α and β across an ensemble, there will be many subensembles $N_{M}$ that can be selected satisfying these constraints. In the remainder of this section, we demonstrate selection of such subensembles, and show as an unavoidable consequence of the underactuated input that their size is limited. In the next section, we consider control of $N_{M}$ as a measure of and a proxy for selective activation across the full, large ensemble.

**Figure 5:**
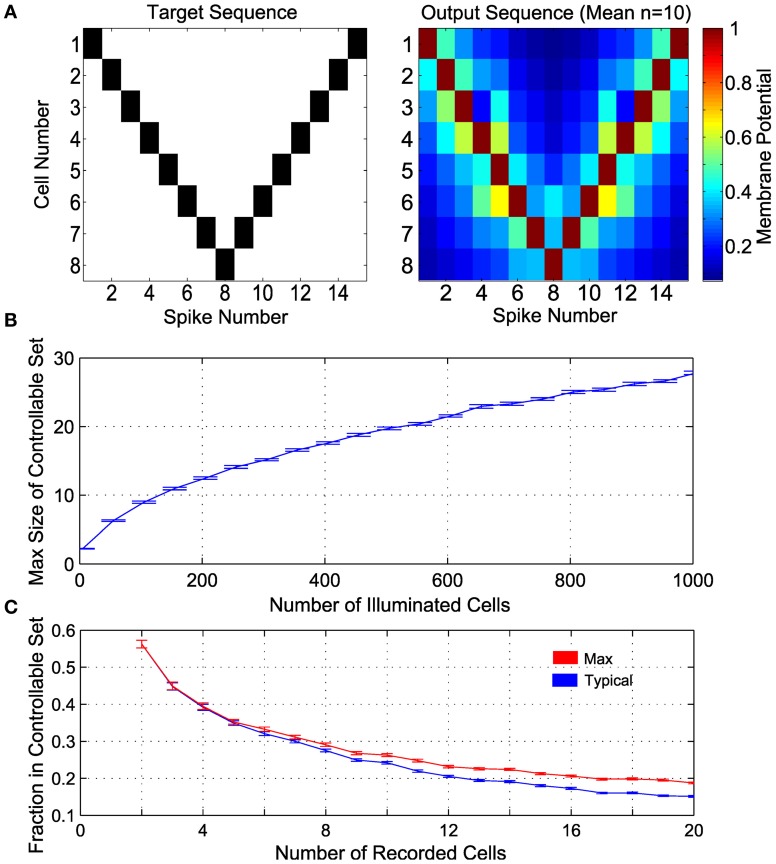
**Control in a population of neurons with random distribution of (β, α). (A)** Control of 8 cells. The target spike sequence (*left*) is used to control *n* = 10 randomly generated ensembles of 8 controllable cells. The mean potential at the time of each spike is depicted on the right. **(B)** Maximum number of controllable cells in an ensemble of 1000 cells, given by the size of the largest subset satisfying the monotonicity criterion. **(C)** Maximum fraction of controllable cells within a random subsample of “recorded” cells (e.g., near an electrode), given by the size of the largest subset satisfying the monotonicity criterion. In addition, a realization of a convex subset is found; this is the Typical fraction of controllable cells. In panels **(B)** and **(C)**, α is lognormal distributed with mean 1 and variance 0.25, β is exponentially distributed with mean 1, *n* = 100 realizations are sampled at each size, with results plotted as mean ± standard error.

From the recorded set of neurons, we want to find a controllable subensemble, noting that such a subset need not be unique. This process amounts to identifying a monotonic subsequence in the (β, α) plane [satisfying Equation (10)], for which standard algorithms are available (Fredman, 1975) (see also Methods). We demonstrate the principle of ensemble control with several realizations of 8-cell controllable subensembles, found with the largest monotonic sequence algorithm, from random draws of a larger ensemble of 100 neurons, when the parameters (β, α) are drawn from exponential and lognormal distributions, respectively (see Methods). We then induce the example spike sequence shown in Figure 5A (left), using Equation (7) by pairing each controlled neuron with a dummy neuron, such that the line joining them in the (β, α) plane lies to the right of all other neurons (see above and Methods 4.2). The mean output is shown in Figure 5A (right), where the desired sequence is successfully achieved.

An important question then is how large *M* can be. Figure 5B shows the maximum possible size of controllable subensembles in a random population of illuminated cells, as a function of the total number of illuminated ChR2+ cells. For comparison to experimental settings, Figure 5C shows the fraction of controllable cells in a subsample of recorded cells, i.e., the proportion of controllable cells likely in common multi-unit recording sizes. Also shown is this fraction for a typical controllable set returned by a suboptimal numerical algorithm that also satisfies Equation (11) (see Methods). As intuition might suggest, a pair of recorded neurons is expected to be controllable around 50% of recordings. With larger recordings, the number of controllable neurons increases, but as a decreasing fraction of the recorded population size.

For generic random distributions of the parameters (β, α), such subensembles are significantly smaller than the ensemble as a whole. We stress that this result is an inherent consequence of the experimental situation rather than the control approach *per se*. Thus, finding controllable sets could require recording from a large number of neurons. Nevertheless, the result shows that, in principle, a measure of spike pattern control in underactuated ensembles is possible. More importantly, controllable subensembles provide a structure by which to determine which other neurons within the full ensemble are impacted by our control inputs. For example, for a desired spike in a given cell, it might be possible to construct a control solution that minimizes the number of extraneous spikes in other cells in the ensemble. Thus we retain some control of the ensemble beyond synchronous bulk activation, as we show next.

### 2.7. Large ensemble response to subensemble control

Any spike induced in a controlled neuron will induce a certain number of collateral spikes in other neurons within the broader illuminated population. We term these neurons as *participating* with the controlled neuron. They corresponded to neurons that lie to the right of the line corresponding to the target neuron's activation.

**Figure 6:**
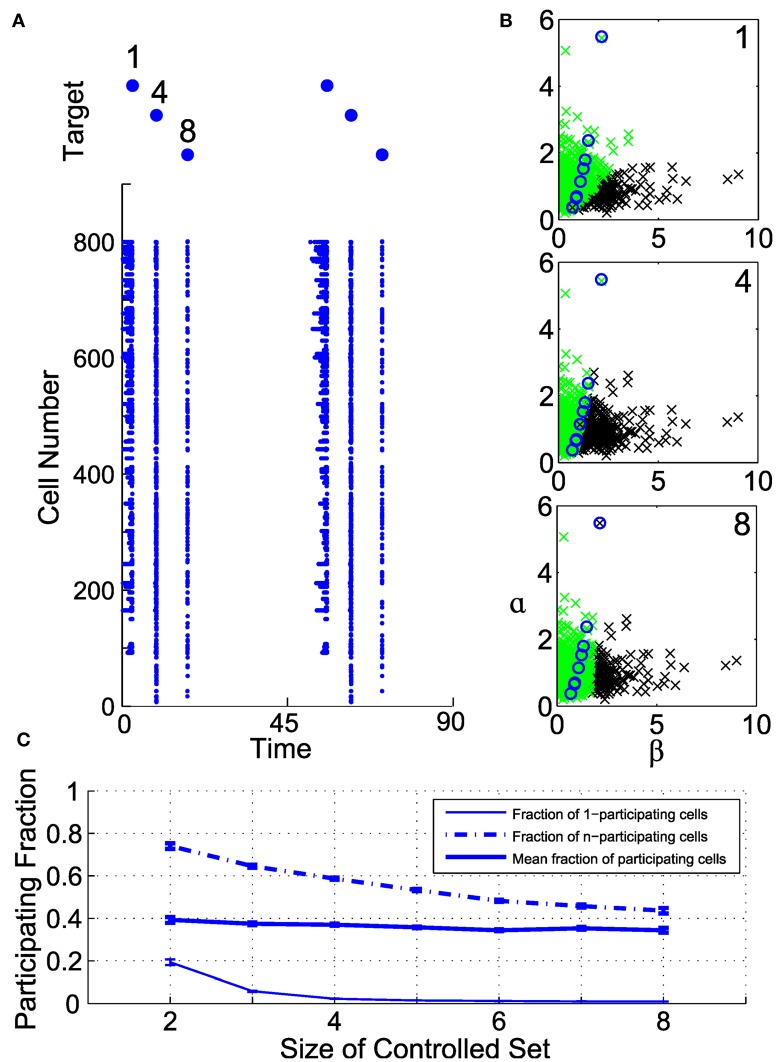
**Geometry of collateral spikes in participating subsets.** (**A**, *top*) A target sequence in three out of a set of eight controlled cells. (**A**, *bottom*) Raster of corresponding spiking in superset of 800 cells. **(B)** Participating sets for controlled spikes, shown in the (α, β) plane. From top to bottom, spikes are driven in cells 1, 4, or 8. Black denotes a participating cell (i.e., a cell that produces a collateral spike), green denotes a non-participating cell, and circles mark cells in the controlled subset. **(C)** Fraction of participating cells as a function of number of controlled cells (see text for definitions).

Figure 6 illustrates the organization of participating cells in an example controllable subensemble of eight cells, sampled from a superset of 800 illuminated neurons. We induce a repeating sequence of spikes in neurons 1, 4, and 8, and show the collateral spiking activity in the full ensemble as rasters in Figure 6A. Each induced spike is associated with distinct but overlapping sets of participating cells. The geometry of these participating sets in the (β, α) plane is shown in Figure 6B. Circles indicate the controlled set of eight cells. Those cells with sufficiently high ChR2 conductance and long membrane time constants (low α) participate with every controlled cell. Similarly, cells with sufficiently low ChR2 conductance and short time constants never participate with any cell. Those cells with intermediate parameterizations may participate with one or more cells, depending on their proximity to and the geometry of the controlled subensemble. Even where the participating sets do overlap for different controlled neurons, the level of activation differs depending on which cell is controlled; note from Figure 6A that the participating set for neuron 1 produces repetitive spikes due to the relatively larger input required to activate that neuron.

To elucidate this further, we examine the fraction of participating cells in a large illuminated ensemble of 2000 neurons, for different size controlled subensembles, constructed from randomly drawn subsets of recorded neurons. Figure 6C shows three quantities related to this fraction as a function of controlled set size. Plotted are the fraction of participating cells that participate with only one of the controlled cells (termed 1-participating), those that participate with all of the controlled cells (termed n-participating), and the average fraction of neurons that participate with at least one controlled cell. Clearly, as the number of controlled cells increases, the fraction of 1-participating cells rapidly decreases. However, so does the fraction of n-participating cells, meaning that only a segment of the illuminated ensemble will always produce collateral spikes. On average, for these parameters, about 40% of neurons participate to some degree with the controlled subensemble, but the size of the participating set depends only weakly on the subensemble size.

This result demonstrates that control of even a few recorded cells will provide a measure of control over a larger ensemble. Hence, we are not resigned to synchronous bulk activation of the ensemble. One could construct control solutions that partition or segment the ensemble into groups, based on the probable location of recorded cells in the parameter space. With sufficiently refined estimates of the distributions of (β, α), one could alternatively maximize the partitioning of the ensemble, without specifically seeking a completely controllable subensemble. If we assume the recorded neurons are a random sample of the full population, we could estimate the likely levels of activation and overlap from control applied to the recorded neurons, as a proxy for the full ensemble. The larger the number of controllable cells, the greater number of groups and control granularity within the ensemble are possible.

## 3. Discussion

Our goal has been to consider large ensemble properties in a “pessimistic” control regime we believe applies to many neurocontrol applications in chronically implanted animals. As such, it is necessary to lay out the simplifying assumptions and limitations of the current analysis, and suggest their likely impacts. We first put our approach in the context of previous work in neurocontrol.

### 3.1. Previous work

The application of control methods in neuroscience is a growing area of interest (Lebedev and Nicolelis, 2006; Grill et al., 2009; Schiff, 2009; Ahmadian et al., 2011). Controlling neuronal spiking has most frequently been studied in the context of electrical stimulation. Significant study has been directed toward global network properties of phase oscillator models of neurons. For example, the objective in (Danzl et al., 2008, 2009; Nabi and Moehlis, 2012) is finding inputs that alter the rate or synchronization of neural populations while satisfying stimulation constraints such as charge balance and minimum current flow. A similar paradigm has been studied in conductance based models, where a non-linear Kalman filtering approach was used to obtain complete state estimates of a single neuron (Ullah and Schiff, 2009). In an alternative approach, (Ahmadian et al., 2011) used a statistical framework to control the probability of spiking assuming either an electrical or optical input. Importantly, these works assume each neuron receives an independent channel of control input.

Less work has been performed on the underactuated case. Effort again has centered on the problem of controlling not the spiking but the overall level of synchronization within a cell population (Tass, 2002, 2003a,b; Nabi and Moehlis, 2011). The recent work (Dasanayake and Li, 2011; Zlotnik and Li, 2012) uses a non-linear methodology derived from quantum spin control to obtain optimal current inputs to control populations of phase oscillator neurons. The solutions allow for setting different spike frequencies in different cells with a single input and are optimal according to classical control theoretic criteria. However, since they arise from oscillator models, the resulting spike sequences are periodic. Moreover, the control is allowed to take both positive and negative values, which may not be available in common experimental paradigms.

From a theoretical standpoint, the integrate and fire framework we have used amounts to a bilinear control problem, where the input is affine to the state (in our case, the membrane potential). Such systems have been well-studied, and certain controllability properties characterized (Tarn et al., 1973; Elliott, 2009). However, the control of such systems with reset dynamics and positivity constraints on the input has not, to our knowledge, been studied. The formulation here differs from the above works by considering control of arbitrary spike sequences, using a non-negative conductance input in a non-periodic setting. We do not explicitly consider a control objective function or prove optimality. Our aim is to identify conditions and control inputs that are viable in current experimental implementations, where measurement is coarse and actuation is broad, and directly construct strategies through analysis of the dynamics.

Our approach depends heavily on the existence of heterogeneity between neurons. Several lines of evidence across physical and biological systems suggest the importance of variation in understanding the function of complex systems (Marder and Taylor, 2011; McDonnell and Ward, 2011). Nervous systems naturally exhibit cell to cell variability, and relying only on mean values of measured parameters can be misleading in predicting circuit function. For example, some intrinsic membrane properties of olfactory bulb mitral cells covary according to whether the cells innervate the same or different glomeruli (Angelo et al., 2012) with strong impacts on firing properties (Angelo and Margrie, 2011), while heterogeneity across mitral cells increases the informativeness of output spike patterns by reducing redundancy (Padmanabhan and Urban, 2010). In other systems, covariation of properties across neurons can result in a fixed overall network behavior over a range of parameter sets, producing a robust output despite cellular variation from animal to animal (Grashow et al., 2010) [reviewed in Marder and Taylor (2011)]. In contrast, heterogeneity across neurons can also constrain network behaviors, such as in the degree of synchronous oscillation (White et al., 1998). Our results focus on exploiting heterogeneity for a neuroengineering control objective, rather than considering the role of noise in normal neural function, but are closely connected to these prior observations. For example, in the simulation of large ensembles (Figure 6) we assumed that alpha and beta vary independently. If there was some homeostatic or other mechanism that resulted in non-independence of parameter variations across cells, the efficacy of an underactuated control scheme would depend on the details of the parameter distributions. For example, if α and β were inversely proportional, fewer pairs would satisfy the condition Equation (3). Conversely, if α and β were positively correlated, more pairs would be controllable. However, only in the case of a convex relationship (e.g., α as a parabolic function of β) would we get a significant increase in the size of the maximally controllable ensemble. Most likely, real neural networks lie somewhere between these extremes.

There are also well-known phenomena for which small amounts of dynamical noise can be beneficial. In the classic example of stochastic resonance (SR), small noise added to a thresholding device allows subthreshold signals to be detected on the output (Tessone et al., 2006; McDonnell and Ward, 2011), a phenomenon seen also in neural networks (Gai et al., 2010; Kawaguchi et al., 2011). In certain settings, such as signal transmission through the summed output of a parallel array of thresholding devices, uncorrelated noise across devices also can improve superthreshold signal transmission (Stocks, 2001) (for an implementation with IAF neurons, see Hoch et al., 2003). It is unclear how addition of independent dynamical noise compares to inclusion of parameter heterogeneity, for example by allowing devices to have different thresholds (Stocks, 2000). In at least some cases, transmission enhancement through dynamical noise is optimal when all devices are identical, and this is the case most widely studied (Stocks, 2001; McDonnell et al., 2007; Kawaguchi et al., 2011). However, some have argued the above examples are just specific instances of a more general phenomenon of “diversity-induced resonance” (Tessone et al., 2006). These ideas remain under investigation. An essential difference from these studies and our results is that, while in all cases a common signal is applied across units, the quality metric employed for SR is signal transmission of a summed output, thereby eliminating the individuality of each unit. Here, we seek specifically those common inputs that diversify the outputs, not for the preservation of signal transmission, but directly to activate subensembles within a region. In applications, the distinction is likely to matter when there are other identities to the neurons, for example tuning to particular sensory events. In the above theoretical results, all neurons were identical and there was no difference (e.g, to downstream areas) in having one or another neuron spike.

### 3.2. Limitations and extensions

We believe many important neurocontrol problems can have pessimistic outcomes from the perspective of traditional control theoretic formulations, and have attempted instead to better determine what control may be possible under the limitations imposed by the experimental structure. While neural systems can present complicated, non-linear dynamics, and previous work has approached control under these dynamics (McIntyre and Grill, 1999; Liu et al., 2010, 2011; Ahmadian et al., 2011), we believe a more fundamental issue is the nearly continuous structure of neural tissue. We are motivated in particular by cortical tissue, where the number of neurons in a cortical column is large enough and the columnar structure rich enough (Meyer et al., 2010; Oberlaender et al., 2012) that it is unclear how much utility there is in deriving control strategies at the single neuron scale. As a nearly continuous substance with fixed substructure (e.g., the topology of synaptic connections, cortical lamination), neural tissue presents control problems fundamentally different from the control of mechanical devices with discrete degrees of freedom that motivates much of the classical theory. For example, it appears that performance may degrade smoothly as the number of controlled neurons decreases, such that relatively crude stimulation to sensory areas can induce perceptual biasing (Romo et al., 2000; Hanks et al., 2006). Neurocontrol problems fall somewhere between the discrete degrees of freedom and independent actuators common in robotics and aerospace control, and well mixed bulk solutions such as in chemical process control.

In cortical tissue, the most widely employed interface for recording and stimulation consists of one or more microelectrodes. We can crudely assess the degree of underactuation as follows. Each contact records from and can stimulate (within safe levels) a diameter of order 100 μm, leading to a coarse estimate of up to a few hundred stimulated cells per contact (Gold et al., 2006; Grill et al., 2009; Histed et al., 2009; Buzsáki et al., 2012). Even neglecting fabrication challenges, the maximum density of electrodes that can be placed within a given volume is limited by the tolerable tissue damage. Imagining an optimistic situation of a MEMS fabricated shaft spanning the cortex, with linearly spaced contacts at high density (10–100, the ratio of total neurons in a column [order 10^4^, (Meyer et al., 2010)] to the number of independently stimulated “pools” could still be of order 100 to 1 or greater. The lateral density of multiple shafts is similarly limited at scales up to 1 mm by concerns about tissue damage, and in general it appears difficult to lower this ratio with implanted devices. This estimate ignores further neural complications such as cortical structure and cell types, but also strategies for current steering using multiple contacts simultaneously.

Optogenetics offers a number of advantages as a stimulation modality. Because stimulation is optical, it is not as critical to place physical contacts close to the desired stimulation site, and because the mechanism of stimulation is through membrane bound proteins and not extracellular current, there may be little to no collateral stimulation. The latter property allows investigation of specific, targeted cell populations, and may also alleviate unintended effects such as direct recruitment of inhibition with electrical microstimulation (Butovas, 2006; Tehovnik, 2006; Histed et al., 2009). A lesser noted property is the ability to stimulate large numbers of neurons in bulk with broad illumination, with the lateral spread of light controlled by the size of the fiber. The scales at which neurons can be activated are determined by the scattering of light in tissue, the irradiance thresholds for activating optogenetic molecules, and maximal illumination before incurring tissue damage, heating, or other undesired effects. No currently conceivable technology for chronically implanted animals would provide an independent light source for every neuron. In awake animals it is difficult to create high illuminator density, so most studies use single sources with broad illumination (Zhang et al., 2009; Anikeeva et al., 2011; Siegle et al., 2011; Royer et al., 2012); we have considered this simplest case. The single input might be thought to allow only bulk activation and synchronous spiking; we have shown here that this is not fully the case. In behavioral experiments, the usefulness of a control strategy such as presented here will depend on the number and composition of participating neurons, coactivated by stimulation of at least one controlled neuron (Figure 6). In the presence of a population of neurons activated by every input, the functional import of differential control of a potentially small subset of neurons is a central open question, likely dependent on the behavioral context and region being stimulated. Several groups have developed multisource systems, e.g., (Zorzos et al., 2010), and in some cases light fields may partially overlap, creating opportunities for multisource control. In this case the model could be modified with, e.g., two inputs β_*a*_ *g*_*a*_(*t*) + β_*b*_ *g*_*b*_(*t*) to each cell, where the difference in β's encode location relative to sources. Follow up work could consider multi-source activation, for example to seek optimal configurations given a “cost per fiber” and a control objective. Analogous to current steering in multielectrode arrays, use of a dynamical strategy such as presented here might achieve a significant improvement in control resolution (e.g, number of controlled vs. participating neurons) with a modest increase in the number of inputs, even where the ensemble remains highly underactuated.

We can further distinguish three broad categories of caveats to our analysis, relating to the degrees of dynamical freedom, the parameter choices, and the state estimation and control strategies, which we discuss in turn.

#### 3.2.1. Dynamics assumptions

The most severe dynamical limitation in the current analysis is the choice of the integrate and fire model. Although this model is analytically convenient and widespread, it cannot represent some phenomena found in biological neurons and higher order models, such as bursting (Breen et al., 2003), intrinsic subthreshold oscillations (Manor et al., 1997; Khosrovani et al., 2007), or rebound spiking (Sohal et al., 2006). There are at least two major mathematical properties of the IAF that could limit broad applicability. The first is that the model is nearly linear; the spike generation mechanism has been compressed into an external and discontinuous reset when the voltage crosses a threshold, and the model has linear (technically, affine) subthreshold dynamics. The second property is that the model is one dimensional. Our approach depends in large measure on the monotonicity of the membrane potential response to the optogenetic drive. As a particular counterexample, harnessing subthreshold inactivation of sodium channels in a Hodgkin–Huxley type model could allow a less excitable cell to overtake a more excitable cell after a set of prepulses (data not shown). Such control strategies are not available in IAF models; however, a control strategy dependent on differential inactivation of ion channel species is more complicated than that presented here, and would require detailed knowledge of channel properties and subthreshold voltage, not readily available from extracellular recordings. To the extent subthreshold dynamics are nearly linear for fast inputs, our simple approach may be viable with little modification, as we demonstrated in Section 2.5.

As a first analysis, we included no synapses or network effects in our formulation. Inclusion would open up new control possibilities, but with substantially greater complication. It may be that connected networks may admit larger controllable sets. In particular, neurons unreachable from direct stimulation might still be activated through appropriate network interactions. However, it would be necessary on a case by case basis to know the details of present cell types (at least inhibitory versus excitatory, but probably a finer categorization), their synaptic topology, and geometric arrangement (de Kock and Sakmann, 2009; Meyer et al., 2010; Oberlaender et al., 2012). It is unlikely that a single general control approach will apply to all network structures, and it is unknown how much improvement could follow from explicit modeling of synaptic interactions.

Other dynamical limitations include a superficial treatment of channel and charge kinetics. We used point neurons, lacking any description of spatial spread of charge or ChR2 channels. We did not include ChR2 kinetics, which exhibit history dependent responses, maximal open and close rates, and possibly low continuously open conductance (Yizhar et al., 2011). Even ignoring channel kinetics, ChR2 conductance should be not only non-negative, but also bounded below some maximal value, determined by expression levels, saturation of the channels, and the maximal illumination the experimenter is willing to apply. In experiments, *g*(*t*) may not be approximated well as directly proportional to the applied light, and perhaps would be replaced by a sigmoidal or other saturating mapping of light to conductance. Many variants of ChR2 are available (Fenno et al., 2011; Yizhar et al., 2011), and in an experimental setting the dynamics of the variant should play a role in the control approach. We note that these assumptions were required, practically if not theoretically, in other recent control theoretic studies incorporating ChR2 (Ahmadian et al., 2011).

#### 3.2.2. Assumptions on parameters

Accepting these dynamical simplifications, there are still non-trivial choices for parameters. Given the choice of IAF dynamics, we further simplified the problem by assuming noiseless neurons and a stable rest point. As a consequence, the ensemble dynamics are determined completely by the parameters (β_*k*_, α_*k*_), allowing us to derive simple conditions on pairwise controllability. This fit our goal of using simple models to try to understand large ensembles, but with more realistic dynamics other strategies may be possible. We assumed both *E* and the threshold *v*_*T*_ are constant across cells. One but not both of *E* or *v*_*T*_ can be scaled away through an appropriate choice of units for membrane potential *v*_*k*_. A consequent change to (β_*k*_, α_*k*_) could be absorbed into their assumed distributions, and thus it is sufficient to consider random distributions on (β_*k*_, α_*k*_, *v*_*T*;*k*_). Small variations in *v*_*T*_ should not change the qualitative results, but further work is required to assess any quantitative effects. Relative to the values of rest and threshold, the ChR2 reversal potential *E* we used in simulations may be low, although it is hard to determine “true” values in such a simple model. The effect of high *E* would be to linearize the dynamics, so that the conductance input is effectively a current, since then *g*(*t*)β_*k*_(*E* − *v*_*k*_) ~ *g*(*t*)β_*k*_ *E* for all subthreshold potentials. The likely effect of this linearization is to relax the conditions for controllability since, in effect, the input is free from the membrane potential *v*_*k*_.

#### 3.2.3. Assumptions on state estimation

We made additional limiting assumptions to the observation model and allowable control inputs, inspired by our experimental context (Zhang et al., 2009; Anikeeva et al., 2011; Siegle et al., 2011; Yizhar et al., 2011). Our approach amounts to an open-loop, feedforward control solution, as we do not process extracellular recordings in real time. In practice, any attempt to apply such a solution would require good estimates of (β_*k*_, α_*k*_). This raises the need for a system identification process prior to initiation of control. Any errors in the resulting estimates would, of course, diminish the accuracy of the derived control input, and in cases where the two cells cross threshold close to each other, would increase the error rate for induced spikes. In Section 2.5 we demonstrated a degree of feasibility by fitting the IAF model to a noisy, multiconductance neural model, using only the spike times. Our open loop approach is most appropriate for situations where we cannot precisely measure the membrane potential, such as in extracellular recording. While methods exist for estimating such potentials, and other variables such as ion channel conductances, from discrete spike times, they tend to be computationally intensive and introduce an additional set of complications (Koyama and Paninski, 2009; Paninski et al., 2009; Meng et al., 2011). Other approaches abstract the biophysics and try to capture the timing of spikes relative to inputs and spiking history without explicit dynamical representation, such as general linear models (Truccolo et al., 2005; Lawhern et al., 2010). Future work could evaluate if fitting such models facilitated more accurate control designs in experimental settings. The IAF is likely to be biophysically inaccurate for many recordings, but provides a more direct path to determining conditions and inputs for sequence controllability, and can perform surprisingly well at fitting and predicting spike trains from real neurons (Gerstner and Naud, 2009). Moreover, the central issue of underactuation would persist, and we would still need to determine how the full ensemble activity relates to that of the smaller controlled set.

Noise arises in several ways, adding uncertainty to the trajectories, and making the control objective probabilistic. We do not expect, at least for small noise, to see a large impact on our ensemble properties such as the controllable fractions, since such noise will be averaged out over the large population. However, the error rate in individual spike trains would inevitably increase. More importantly, noise in the form of spontaneous activity could weaken our assumption that neurons return to their resting state after each spike. Of course, if there is a strong structured input from any source other than our illumination, no feedforward control strategy could result in dependable spike patterns, and a more challenging closed loop control strategy would be needed (Ahmadian et al., 2011). However, under reasonable assumptions of small and unstructured spontaneous membrane potential fluctuations, our control strategy could apply to a “ball” of initial conditions around rest, for which there is only small deviation of trajectories during the fast approach to spiking.

Relying on cells to return to rest after each spike constrains the timing of controlled spikes to the scale of membrane time constants. Measured time constants typically fall within 10–100 ms, with most estimates falling near 20 ms (Koch et al., 1996; Mensi et al., 2012; Varela et al., 2012), including specifically in our area of interest, rodent somatosensory cortex (Feldmeyer et al., 1999; Siegle et al., 2011). Because the time constant depends on the total membrane conductance, it can be significantly shorter (e.g., 5 ms) during states of high input (Mensi et al., 2012). More generally, the effective decay rate may differ from the time constant estimated from passive membrane properties with non-linear models (Koch et al., 1996; Wei and Wolf, 2011). Under our approach, these estimates suggest typical maximal control rates around 10 Hz, with a range from 2 to 40 Hz. Even at the slower end, these rates should be taken in the context of observed spontaneous and evoked rates in cortex, which are often below 1 Hz (Brecht, 2003; DeWeese et al., 2003; Brecht and Sakmann, 2004; O'Connor et al., 2010), albeit with a “heavy tail” toward higher rates. In other words, while extracellular recording selectively samples from neurons with high spontaneous rates, the ability to control spike rates even if limited to 10 Hz represents a substantial modulation of spike rate for a large fraction of neurons. More elaborate strategies that do not require this return to rest could likely operate at higher spike rates, and possibly be more robust to errant spikes, but again would require good estimations of continuous states, based on limited observability through spike times.

#### 3.2.4. Implications of the current results

In the context of these caveats, we now recap our major results and their most likely impacts for more realistic models and in experiments. First, we have provided analytic results in a simple model that establish the feasibility of using a single input (optical fiber) to achieve a degree of independent spike control in pairs of neurons. We extended this result to consider control in large ensembles, showing that some degree of independence is possible beyond synchronous activation of a fixed set of neurons. The approach could be readily implemented in experiments involving recording of single unit activity simultaneously with application of optogenetic stimulation. A prominent remaining hurdle is the system identification step for finding (β_*k*_, α_*k*_). While not considered here, a number of empirical approaches could be used for this purpose (Koyama and Paninski, 2009; Paninski et al., 2009; Meng et al., 2011). The testing of our method in an experimental preparation is the subject of ongoing work. Our results are a first step in the development of control solutions for expanding control of ensemble spiking beyond synchronous activation, in the underactuated situations we see as typical given available stimulation technology for the near future.

## 4. Methods

### 4.1. Numerical implementation

All simulations were implemented in the MATLAB (Mathworks, Inc.) environment using standard Euler or Runge–Kutta methods for solving ordinary differential equations. The Euler step size was chosen as 0.002. The non-linear reset operation was implemented using a threshold of *v*_*T*_ = 1. After each spike, membrane potential was reset to *V*_*reset*_ = 0.0001. When large neuronal ensembles were randomly generated, the parameter α was drawn from a lognormal distribution of mean 1 and variance 0.25, while the parameter β was drawn from an exponential distribution of mean 1. These distributions were chosen loosely based on experimental and biophysical intuition, assuming that leak conductance must always take a finite value (i.e., α > 0), while certain cells may have little to no ChR2 expression (i.e., β ≥ 0). The MATLAB functions exprnd and logrnd were used to sample these distributions.

### 4.2. Algorithm for control of multiple cells

The following algorithm was used to select inputs for a given set of controllable cells (e.g., Figure 5A). Assume that the subensemble is labeled as in Equation (11) for *i* = 1, …, *N*. For a desired spike in cell *N*_*i*_:

- If *i* ≠ 1 and *i* ≠ *N*, calculate *s*_*i*_ = (α_*i*_ − α_*i* − 1_)/(β_*i*_ − β_*i* − 1_) and *s*_*i* + 1_ = (α_*i* + 1_ − α_*i*_)/(β_*i* + 1_ − β_*i*_). Take s_*_ as the mean of *s*_*i*_ and *s*_*i* + 1_.
- If *i* = 1, s_*_ = (*s*_2_ + (α_1_/β_1_))/2.
- If *i* = *N*, s_*_ = *s*_*N*_ × 2.
- Calculate
  $$y_{*}=\alpha_{i}-s_{*}\beta_{i}.$$
- Create a “dummy” cell *N*_*D*_ with parameters along the line in the (β, α)-plane defined by slope s_*_ and α-intercept y_*_.
- Apply control input Equation (7) for *N*_*i*_ and *N*_*D*_.

By construction, a spike is achieved in *N*_*i*_ and all other cells in the controlled set remain subthreshold by virtue of lying to the left of the line connecting *N*_*i*_ and *N*_*D*_. This algorithm is not optimal (in the sense of minimizing stimulation energy or timing, or maximizing robustness to noise), and is designed to simply demonstrate selective spiking control in the underactuated ensemble.

### 4.3. Simulation of biophysical neurons

In Figure 4, each neuron was simulated using biophysical voltage-gated conductance equations of the Hodgkin–Huxley type. These neurons are based on non-linear equations that describe the dynamics of sodium, potassium, and leak channels, and previously published in (Ching et al., 2010, 2012).

For each neuron, we used a system identification procedure to fit the parameters (β, α) of a corresponding IAF model. While many such procedures could be used, we opted for a fit based on minimizing the difference in spike timing of the biophysical neuron and the fitted integrate-and-fire neuron, upon excitation by pulse inputs. We used the non-linear least-squares function fminsearch in MATLAB to effect the fit. After making the fit for the two neurons, we applied the algorithm in Section 2.3 to find inputs that achieve selective spiking in the two IAF neurons. This control input was then applied to the original HH-type neurons, leading to the output shown in the figure.

### 4.4. Subensemble construction

Figure 5B reports the maximal controllable set size as a function of the size of the illuminated population. At each point on the x axis of this figure, a random realization of corresponding size is created by drawing from the above random distributions of (β, α). From that realization, the length of the longest monotonic subsequence [satisfying Equation (10)] is obtained using a standard algorithm (Fredman, 1975). This length constitutes the maximal controllable set size. Figure 5C reports the maximal and typical controllable fractions. At each point on the x-axis of this figure, a random realization of the corresponding size is created by drawing from the same distributions as in Figure 5B. The maximal controllable fraction is the maximal set size divided by the value on the x-axis. The typical controllable fraction is found by finding a subset that satisfies not only Equation (10) but also the convexity criterion Equation (11). To find a convex set, we used a suboptimal method using repetitive random subsampling of the set and creation of a convex hull using the MATLAB function convhull.

Figure 6 was created by generating random realizations of cell ensembles of variable size. From each ensemble, a controllable set was realized as above. A superset of 1000 cells was then generated and the percentage of cells that produced collateral spikes in that superset was computed for each controlled cell. The average over all controlled cells is then computed. We report this average for 100 realizations of each controlled set size.

### Conflict of interest statement

The authors declare that the research was conducted in the absence of any commercial or financial relationships that could be construed as a potential conflict of interest.
